# Supplementary material for: Probiotic Fermentation of Astragalus membranaceus and Raphani Semen Ameliorates Cyclophosphamide-Induced Immunosuppression Through Intestinal Short-Chain Fatty Acid-Dependent or -Independent Regulation of B Cell Function
Source: Biology (Basel). 2025 Mar 19;14(3):312. doi: 10.3390/biology14030312 (PMC12077259; doi:10.3390/biology14030312)
Supplement: Supplementary file 1 [file biology-14-00312-s001.zip › Figure S1.pdf]

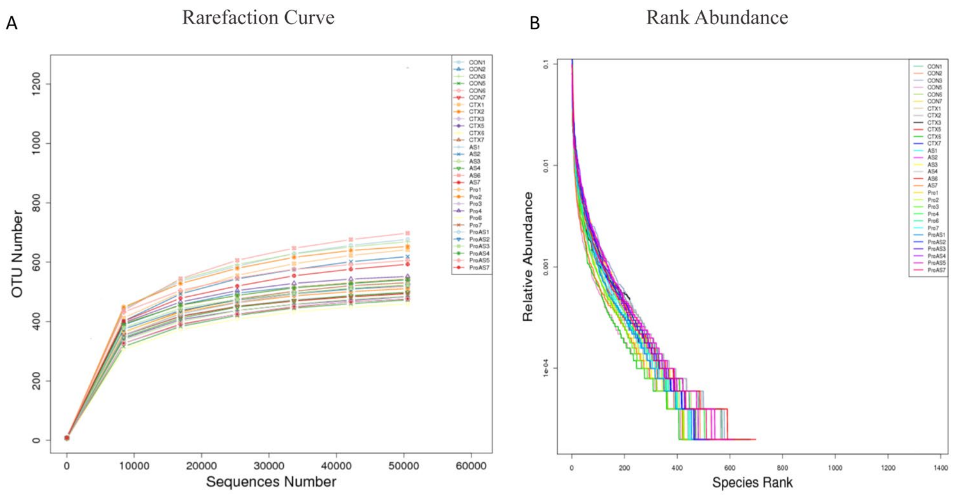

Figure S1. Rarefaction curve and abundance rank curves of 16s RNDA sequencing. **(A)** Rarefaction curve. The curve rises rapidly and then flattens out, indicating sufficient sequencing and complete species coverage; **(B)** Abundance rank curves. The abundance rank curves exhibited a stepwise decline, suggesting the coexistence of multiple dominant species.
