# Supplementary material for: Probiotic Fermentation of Astragalus membranaceus and Raphani Semen Ameliorates Cyclophosphamide-Induced Immunosuppression Through Intestinal Short-Chain Fatty Acid-Dependent or -Independent Regulation of B Cell Function
Source: Biology (Basel). 2025 Mar 19;14(3):312. doi: 10.3390/biology14030312 (PMC12077259; doi:10.3390/biology14030312)
Supplement: Supplementary file 1 [file biology-14-00312-s001.zip › Supplementary Table S1.pdf]

Table S1. Primers used in Quantitative Real-time PCR.

gene	Forward (5'-3')	Reverse (5'-3')	length
Zo-1	CATCATTCGCCTTCATAC	TTGCTTAGAGTCAGGGTTA	142 bp
Claudin-1	TACTTTCCTGCTCCTGTCC	CTCTTCCTTTGCCTCTGTC	112 bp
Claudin-4	CCTTCATCGGCAGCAACA	GGCGAGCATCGAGTCGTA	116 bp
β-actin	AGCCATGTACGTAGCCATCC	GCTGTGGTGGTGAAGCTGTA	222 bp
PRDM1	GTACAAGCTGCCCCCAAGTC	ATGCCTCGGCTTGAACAGAA	202 bp
H2-Aa	GGTGGGCACCATCTTCATCA	GTGTGAGCTGTGAGAGGGAC	241 bp
H2-Ab1	TTTGCTTTCTGAAGGGGGCA	TCGCCCATGAACTGGTACAC	187 bp
